# Functional circuits of new neurons in the dentate gyrus

**DOI:** 10.3389/fncir.2013.00015

**Published:** 2013-02-25

**Authors:** Carmen Vivar, Henriette van Praag

**Affiliations:** Neuroplasticity and Behavior Unit, Laboratory of Neurosciences, Intramural Research Program, National Institute on Aging, National Institutes of HealthBaltimore, MD, USA

**Keywords:** dentate gyrus, adult neurogenesis, rabies virus, retrograde trans-neuronal tracing, learning and memory, area CA3, lateral entorhinal cortex, pattern separation

## Abstract

The hippocampus is crucial for memory formation. New neurons are added throughout life to the hippocampal dentate gyrus (DG), a brain area considered important for differential storage of similar experiences and contexts. To better understand the functional contribution of adult neurogenesis to pattern separation processes, we recently used a novel synapse specific trans-neuronal tracing approach to identify the (sub) cortical inputs to new dentate granule cells (GCs). It was observed that newly born neurons receive sequential innervation from structures important for memory function. Initially, septal-hippocampal cells provide input to new neurons, including transient innervation from mature GCs as well as direct feedback from area CA3 pyramidal neurons. After about 1 month perirhinal (PRH) and lateral entorhinal cortex (LEC), brain areas deemed relevant to integration of novel sensory and environmental information, become substantial input to new GCs. Here, we review the developmental time-course and proposed functional relevance of new neurons, within the context of their unique neural circuitry.

## Introduction

The hippocampus, a brain area important for the acquisition of new memories (Scoville and Milner, 1957; Squire et al., 2004), consists of three subfields: dentate gyrus (DG), area CA3 and area CA1. Information is considered to be processed from entorhinal cortex (EC) to DG, DG to CA3 pyramidal cells, and from CA3 to CA1 pyramidal cells to be ultimately stored in cortex, forming the “tri-synaptic hippocampal circuit” (Amaral and Witter, 1989). Each of these regions has specific cell types and plasticity contributing to learning and memory processes (Nakazawa et al., 2002, 2004; Gold and Kesner, 2005; Kesner, 2007). The DG is of particular interest as new dentate granule cells (GCs) are generated continuously in the adult mammalian brain (Altman and Das, 1965; Cameron and McKay, 2001; Ming and Song, 2011). Over the past decade, the maturation, integration into the hippocampal network, and the functional relevance of new GCs has been researched extensively (for review see Zhao et al., 2008; Suh et al., 2009; Deng et al., 2010; Ming and Song, 2011). Adult neurogenesis is considered important for regulation of cognition and mood (Zhao et al., 2008), and has been proposed as a mechanism underlying efficient cortical storage of new memories (Kitamura et al., 2009). It has also been suggested that new neurons contribute to pattern separation (Clelland et al., 2009; Creer et al., 2010; Guo et al., 2011; Sahay et al., 2011; Nakashiba et al., 2012), the distinct encoding of very similar events or stimuli, a function attributed to the DG (Marr, 1971; Gilbert et al., 2001; Leutgeb et al., 2007). However, until recently, relatively little was known about the specific circuitry into which the new neurons are integrated, which may provide further clues to their functional role. Using a novel combination of viral vectors (Vivar et al., 2011, 2012), we found that newborn neurons have unique afferents. In particular, new GCs receive inputs from mature GCs, a direct “back-projection” from area CA3 and predominant innervation from the lateral (LEC) rather than the medial (MEC) entorhinal cortex. LEC and MEC provide different types of information to the hippocampus (for review see Knierim et al., 2006; McNaughton et al., 2006; Lisman, 2007). Stronger input from LEC (information about external cues and context) than from MEC (spatial position information) may facilitate the role of newly born neurons in pattern separation. The potentially important role of new neurons in this process as well as the time-course of their physiological and anatomical integration into the hippocampal circuitry is the focus of this review.

## Methods relevant to adult neurogenesis

**Figure 1:**
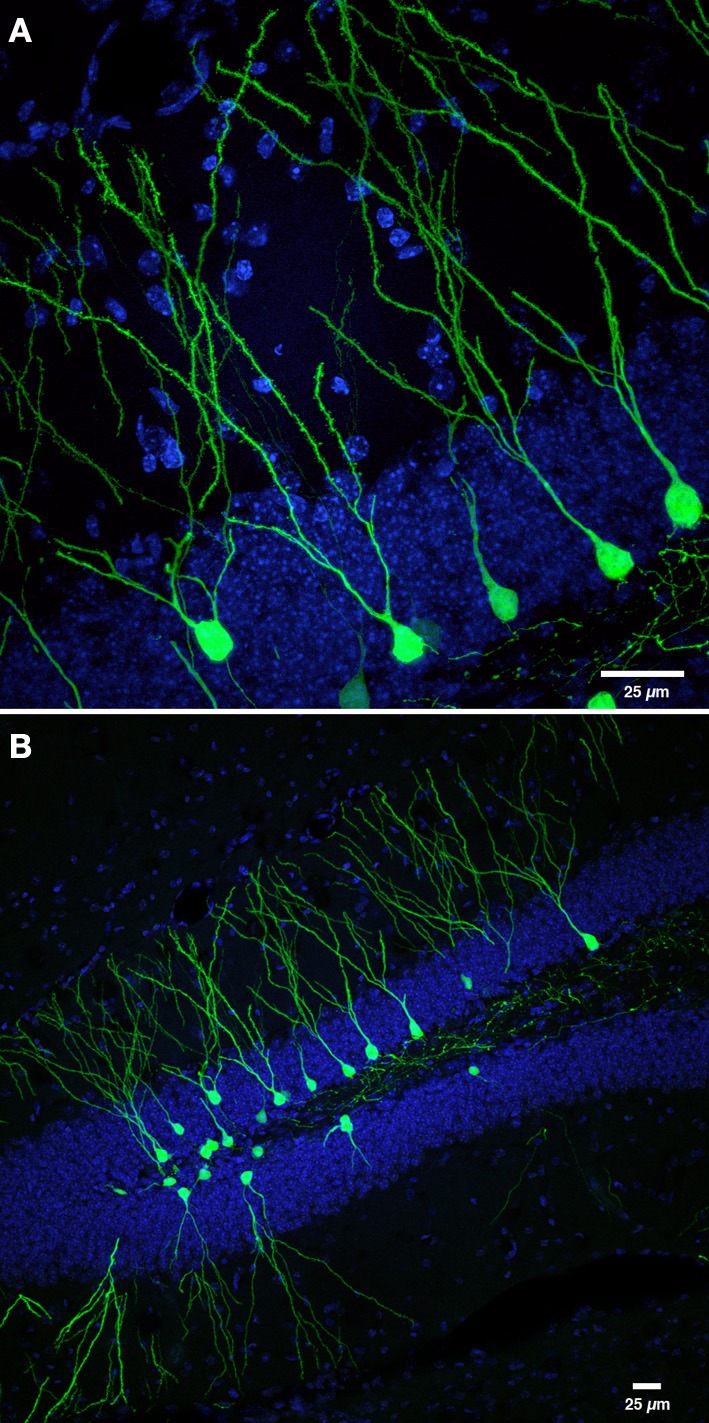
**Retroviral labeling of newborn neurons in the DG. (A)** Photomicrograph shows retrovirally labeled newborn dentate GCs (pCAGGFP, Zhao et al., 2006) expressing green fluorescent protein (GFP) throughout the entire cell, at 42 days post-retroviral injection (dpi). **(B)** Low power overview of new neurons shown in panel **(A)**, expressing cytoplasmic GFP in the DG at 42 dpi. Nuclei are labeled with 4′-6-diaminodino-2-phenylindole (DAPI), blue. Scale bar, 25 μm.

The initial studies that suggested the adult brain could generate new neurons were largely ignored. In the 1960s Joseph Altman and colleagues used tritiated thymidine autoradiography to label dividing cells, but could not prove conclusively that these were new DG neurons rather than glia (Altman and Das, 1965). Subsequently, combined electron microscopy and tritiated thymidine labeling was used to show that labeled cells in the rat DG have ultrastructural characteristics of neurons, such as dendrites and synapses (Kaplan and Hinds, 1977). An important advance was the use of the thymidine analog, 5-bromo-2′-deoxyuridine (BrdU), which is incorporated into the genome of dividing cells, and can be combined with specific neural markers (Kuhn et al., 1996). Retroviral methods, selective for dividing cells (Figure 1), can be used for birth-dating, genetic marking, electron microscopy, and electrophysiology, and have provided strong evidence that newborn neurons in the adult mammalian brain are functional and synaptically integrated (van Praag et al., 2002; Carleton et al., 2003; Ming and Song, 2005). Furthermore, modulation of neurogenesis using x-irradiation, pharmacology, environmental factors, and transgenic mouse models has provided important functional insights about the possible role of these new neurons in the adult brain. Reduction of neurogenesis generally results in deficient memory function whereas increased cell genesis is associated with enhanced cognition (for review see Zhao et al., 2008; Deng et al., 2010).

**Figure 2:**
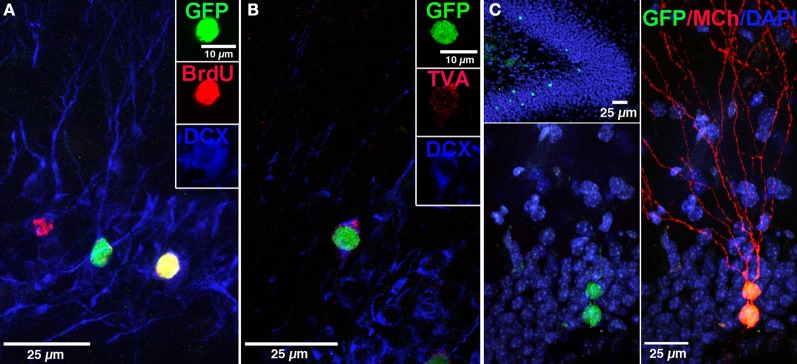
**Labeling of “starter” neural progenitor cells. (A)** Photomicrograph showing retrovirally labeled newborn GCs (pRV-SYN-GTRgp) expressing nucleus-localized histone-tagged green fluorescent protein (hGFP), avian TVA receptor, and rabies glycoprotein under control of the synapsin promoter. Co-labeling of hGFP^+^ cells (green), with bromodeoxyuridine (BrdU, red), and the immature neuronal marker doublecortin (DCX, blue). Insert shows a new GC expressing all three markers (yellow). **(B)** Confocal image of hGFP^+^ cell (green) co-labeled with DCX (blue) and the TVA receptor (red). **(C)** Overview of the DG showing newborn GCs labeled with retrovirus expressing nuclear hGFP (green, top). Magnification of the GC layer shows dual virus labeled newborn “starter” cells with retrovirus expressing hGFP (green) and EnvA pseudotyped rabies virus expressing MCherry (MCh, red) at 30 dpi. Scale bar, 25 μm. Nuclei labeled with DAPI (blue). From Vivar et al. (2012); Reprinted with permission from Macmillan Publishers Ltd.: Nature Communications Copyright 2012.

**Figure 3:**
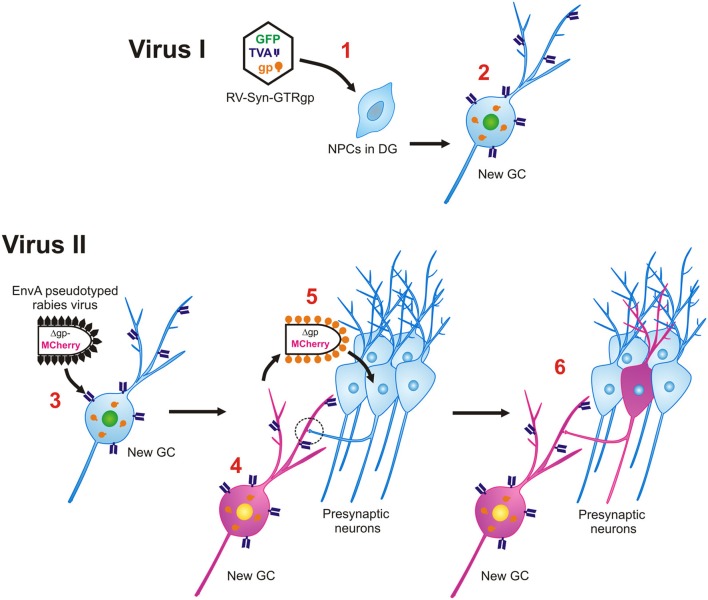
**Monosynaptic retrograde tracing of inputs to new GCs. Virus I, Retroviral labeling. 1**. Retrovirus (RV-SYN-GTRgp) expressing nucleus-localized histone-tagged green fluorescent protein (hGFP), avian TVA receptor, and rabies glycoprotein (gp) under control of the synapsin promoter infect proliferating neural progenitor cells (NPCs) in the DG. **2**. The retrovirally labeled NPCs differentiate into newborn GCs over time and express hGFP, TVA and gp. **Virus II, Rabies virus. 3**. Avian envelope glycoprotein EnvA pseudotyped rabies virus, in which rabies gp was replaced with MCherry (EnvA-ΔG-MCh) is injected into the same DG at different time-points after retroviral labeling (21–90 dpi). Through interaction between EnvA glycoprotein and its receptor, TVA, pseudotyped rabies virus can selectively infect newborn GCs. **4**. EnvA-ΔG-MCh rabies virus is complemented with rabies gp provided by the retrovirus and MCherry is expressed in the cytoplasm. **5**. The rabies virus spreads trans-synaptically to presynaptic neurons connected to the new GC. **6**. Only neurons synaptically connected are labeled and express MCherry. The traced cells lack rabies gp, therefore this virus will not spread any further.

All of the above methods, however, do not reveal the circuitry into which the newborn GCs are integrated. Network analysis is essential for understanding how new neurons are activated, as well as comprehending the functional significance of adult neurogenesis. To overcome this limitation, we recently used a novel combination of retroviral labeling with rabies virus as a retrograde tracer (Figures 2 and 3). Rabies virus as a trans-neuronal circuit tracer offers several advantages over other conventional neuronal tracers (for review see Callaway, 2008; Ugolini, 2010, 2011). Rabies virus propagates by trans-neuronal transfer exclusively in retrograde direction. In particular, intracellular transport of rabies virus after replication is only directed to neuronal dendrites (Ugolini, 1995), and subsequently by retrograde trans-neuronal transfer to presynaptic terminals of higher order neurons (Callaway, 2008; Ugolini, 2010, 2011). Rabies virus propagation occurs at chemical synapses, regardless of their neurotransmitters, synaptic strength, termination site, or distance (Ugolini, 1995, 2010), but not via gap junctions (Tang et al., 1999) or cell-to-cell spread (volume transmission) (Tang et al., 1999; Ugolini, 2010). The mechanisms underlying selective retrograde transport of the rabies virus are not fully understood, however, it has been proposed that several presynaptic elements can act as rabies virus receptors, including the p75 neurotrophin receptor, the nicotinic acetylcholine receptor, and NCAM, among other, as of yet unidentified receptors. The large variety of neurons infected by rabies virus suggests that presynaptic receptors for rabies virus are ubiquitously distributed in the central nervous system (Ugolini, 2010).

Modifications of the rabies virus genome have made it possible to control synaptic spread, reduce pathogenicity, infect select cell types, and add optogenetic tools (Osakada et al., 2011). Trans-synaptic retrograde spread of rabies virus has been proposed to be critically dependent on rabies glycoprotein (Rgp; Etessami et al., 2000). Recently, a glycoprotein-deleted (ΔG) variant of the SAD-B19 strain of rabies virus (SADΔG, Mebatsion et al., 1996) in which Rgp was exchanged for a fluorophore such as green fluorescent protein (GFP) or MCherry (MCh) was developed. Providing exogenous Rgp to infected cells allows the virus to cross one synapse, enabling the selective study of infected first-order afferents (Wickersham et al., 2007a). Further specificity can be achieved by pseudotyping the ΔG rabies virus with an avian viral glycoprotein (EnvA) to selectively infect mammalian neurons modified to express the, typically foreign, avian TVA receptor (Wickersham et al., 2007b; for review see Ginger et al., 2013). Monosynaptic trans-neuronal tracing with rabies virus has been applied to analyze the properties of neural circuits in different parts of the central nervous system such as the amygdala (Haubensak et al., 2010), olfactory bulb (Arenkiel et al., 2011; Miyamichi et al., 2011), visual cortex (Wickersham et al., 2007b; Marshel et al., 2010; Rancz et al., 2011), barrel cortex (Wall et al., 2010), cerebellum (Wall et al., 2010), ventral tegmental area (Lammel et al., 2012; Watabe-Uchida et al., 2012), spinal cord (Stepien et al., 2010), and retina (Yonehara et al., 2011). Recently, Arenkiel et al. (2011) applied the EnvA-TVA methodology to identify inputs to newborn olfactory bulb neurons, the only other brain area considered to generate new neurons under basal conditions (Ming and Song, 2011). Specifically, a conditional reporter mouse was generated, harboring a Cre/LoxP-dependent allele driving cytosolic tdTomato expression upon electroporation of a plasmid containing Rgp, TVA, and Cre, which was introduced on postnatal day 2 into the lateral ventricles. Thirty days later EnvA pseudotyped rabies virus was injected to trace connections to the tdTomato, TVA and Rgp expressing cells. It was shown that early postnatal-born GCs of the olfactory bulb make synaptic connections with cortical inputs and multiple olfactory bulb cell types which could be modified by olfactory experience.

We applied the powerful EnvA-TVA tracing method to map the inputs to newborn dentate GCs by developing a selective and direct dual virus approach that can be used in wild-type animals. Monosynaptic rabies virus-mediated retrograde tracing was combined with retroviral labeling (Vivar et al., 2011, 2012). Specifically, Murine Maloney leukemia virus (MMLV) retrovirus which only infects dividing cells (Lewis and Emerman, 1994; van Praag et al., 2002) was modified to express nuclear GFP, TVA receptor and Rgp. This vector was used to label proliferating neural progenitor cells in the DG that are destined to become neurons by using the neuron-specific synapsin promoter. Double-labeling of GFP^+^ cells with the immature neuronal marker doublecortin in the majority of the labeled cells at 15 days postinjection, supported the specificity of the retrovirus (Figure 2A). Following an interval of 21, 30, 60, or 90 days after retroviral injection during which the progenitor cells matured into GC neurons, EnvA pseudotyped rabies virus (EnvA-ΔG-MCh) was injected into the DG and mice were perfused 1 week thereafter. This rabies virus selectively infects new neurons expressing the TVA receptor, which were termed “starter cells” (Figures 2B,C). The rabies virus is then complemented with Rgp provided by the retrovirus in the new neurons. The virus crosses synapses, labeling presynaptic neurons, and because the traced cells lack Rgp, the virus does not spread any further, labeling only first order inputs (Figure 3). Indeed, when Rgp was deleted from the retroviral vector and subsequent infection with EnvA-ΔG-MCh was performed, double-labeled new GCs (GFP + MCh) were observed but not traced cells, indicating Rgp is required for the system to work. Altogether, this novel approach has allowed us to evaluate how the anatomy of newborn neuron circuitry changes over time and to identify the neurochemical characteristics of their specific inputs. Moreover, as rabies virus does not compromise cell viability, at least for 2 weeks after infection, characterization of the physiology and synaptic plasticity of afferent inputs can be performed (Wickersham et al., 2007a; Callaway, 2008; Vivar et al., 2012).

## Time-course of circuitry development

**Figure 4:**
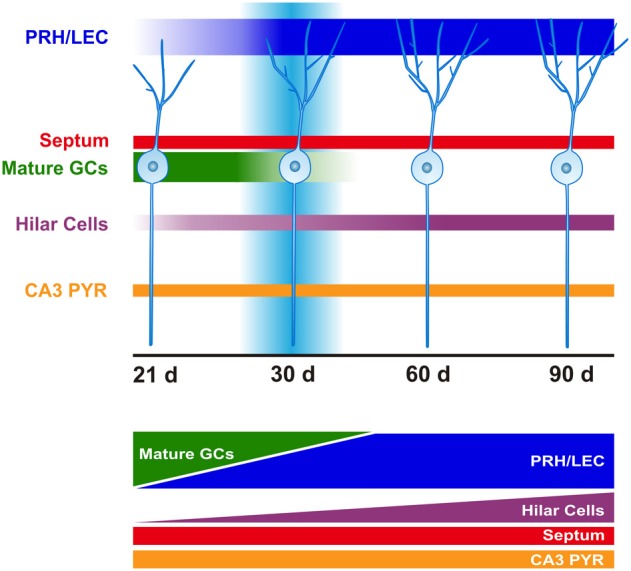
**Time course of afferent innervation of newborn GCs.** Schematic representation of monosynaptic inputs to the newborn GCs at time-points evaluated (21, 30, 60, and 90 days after retroviral infection). At 21 days (d), newborn GCs mainly receive connections from mature GCs. This input gradually shifts from mature GCs to cortex following strengthening of afferents from the perirhinal (PRH) and lateral entorhinal cortex (LEC). Increased hilar cell (mossy cells and interneurons) innervation is observed over time. Convergence of excitatory afferents onto the newborn GCs around 30 days correlates with transiently enhanced new neuron excitability and plasticity. Innervation from septum and area CA3 pyramidal cells (PYR) remains constant over time.

It is generally considered that it takes about 1 month for proliferating progenitor cells to develop into new GC neurons and that full maturation takes several months (Zhao et al., 2008). During the first month, subgranular zone neural stem cells (Type I cells) and progenitor cells (Type II, Type III) have been shown to go through stages with distinct morphological, physiological and molecular characteristics. Newborn neurons are considered to originate from Type I neural stem cells in the subgranular layer. These cells have radial processes extending into the molecular layer, are deemed relatively quiescent and express markers such as nestin, glial fibrillary acidic protein (GFAP) and Sox2. Activation of Type I neural stem cells is considered to be mediated by niche factors such as Notch or bone morphogenic protein (Lugert et al., 2010; Mira et al., 2010) and the neurotransmitter GABA (Song et al., 2012). Type I cells likely give rise to Type II progenitor cells under the influence of additional local niche factors [fibroblast growth factor-2 (Jin et al., 2003), sonic hedgehog (Lai et al., 2003), vascular endothelial growth factor (Cao et al., 2004), and Wnt7a (Qu et al., 2010)]. A subset of the cells retain neural stem cell markers (such as nestin and Sox2), whereas the remaining cells begin to differentiate along a neuronal lineage becoming NeuroD and Prox1 positive, progressing into Type III neuroblasts expressing markers such as PSA-NCAM, calretinin and doublecortin before maturing into GCs (Encinas et al., 2006; Suh et al., 2007, 2009; Lugert et al., 2010). While 1 month later these cells have the morphological and physiological characteristics of GCs, their full maturation and incorporation into functional circuits appears to be a prolonged process. Indeed, newborn neuron physiology, plasticity, and circuitry may continually evolve for at least 3 months (Figure 4).

### First week

During the first week of the maturation process, neuronal lineage-committed Type II cells begin to migrate into the inner GC layer of the DG. Initially, there are no clear dendritic or axonal processes and patch-clamp recordings indicate that the cells do not show spontaneous synaptic activity (Esposito et al., 2005). However, these cells are tonically activated by ambient GABA. Indeed, upon recording from retrovirally labeled cells 3 days after infection a tonic inward excitatory current could be selectively blocked by bicuculline, suggesting non-synaptic activation of GABA_A_ receptors (Ge et al., 2006).

### Second week

During the second week, the cells extend spineless dendrites that reach the inner molecular layer (I-ML) 10 days after retroviral injection (dpi), and the middle molecular layer (M-ML) at 14 dpi. In addition, mossy fiber axons are estimated to begin to form synapses with pyramidal cells at 10–14 dpi (Zhao et al., 2006). The physiological properties of the cells are immature with cells firing few action potentials with a small amplitude (Esposito et al., 2005; Vivar et al., 2012). GABA continues to be depolarizing (Lo Turco and Kriegstein, 1991). However, at this time-point hilar interneurons are considered to provide synaptic input to newborn neurons (Tozuka et al., 2005; Ge et al., 2006). The depolarizing action of GABA depends on the Na-K-2Cl co-transporter (NKCC1). NKCC1 maintains high [Cl^−^]_i_ and regulates the resting membrane potential of developing neurons (Ge et al., 2006; Mejia-Gervacio et al., 2011). A recent study shows that knockdown of NKCC1 *in vivo* reduces DG progenitor cell proliferation and delays dendritic development (Young et al., 2012).

Around this time-point, the cells go through a critical period. Indeed, more than 50% of adult born granule neurons are lost about 2 weeks into the maturation process (Cameron et al., 1993; Dayer et al., 2003). Around 14 dpi, glutamatergic signaling through NMDA receptors may be critical for the survival and integration of newborn neurons (Tashiro et al., 2006). Since the main glutamatergic input to newborn GCs from the EC develops around 1 month (Mongiat et al., 2009), innervation by ipsi- and contralateral mossy cells (Kumamoto et al., 2012) and/or mature GCs may provide the glutamatergic input critical for new cell survival. Morphological evidence for input from mature GCs to newborn GCs was substantiated by electrophysiological recordings performed at short time intervals (1–2 weeks) after viral labeling. Specifically, cells double-labeled for both vectors (retrovirus + rabies virus expressing “starter” cells) showed immature physiological characteristics, while cells with single-labeling (rabies virus only, afferent “traced” cells) exhibited properties of mature GCs in the same acute slice (Vivar et al., 2012), suggesting mature-newborn GC connectivity.

Interestingly, intra-granular connections have been observed after denervation of EC input to the DG, producing sprouting of the mossy fibers into the molecular layer. These mossy fibers form synaptic contacts with spines on proximal dendritic segments of GCs, suggesting that the lack of EC input may be compensated for by intra-granular synapses (Frotscher and Zimmer, 1983). This connectivity has been also observed after seizures or brain injury, producing the same extension of mossy fibers to the molecular layer (Buckmaster et al., 2002; Marqués-Marí et al., 2007; Murphy et al., 2011). It should be noted that seizures accelerate the integration of the new GCs, albeit with a reduction of dendritic length (Overstreet-Wadiche et al., 2006), and increase adult neurogenesis (Parent and Lowenstein, 2002). Whether this can be interpreted as brain self-repair or lead to further pathology (Parent, 2003; Pun et al., 2012; Sanchez et al., 2012) remains to be determined.

### Third week

During the third week, the cells begin to resemble mature GCs more closely. The GC dendrites reach the outer molecular layer (O-ML) with spine formation from 17 dpi onwards. In addition, mossy fiber output to area CA3 increases (Zhao et al., 2006), physiological properties are more mature and spontaneous synaptic activity is detected, indicative of synaptic input (Esposito et al., 2005; Vivar et al., 2012). Furthermore, GABA has become hyperpolarizing and excitatory glutamatergic responses are consistently observed (Ge et al., 2006; Ming and Song, 2011). The source of excitatory input, according to our observations, is mainly from mature GCs, hilar mossy cells, a direct “back-projection” from area CA3 pyramidal cells and sparse input from LEC and perirhinal cortex (PRH) (Vivar et al., 2012). Interestingly, the direct “back projection” from area CA3 pyramidal cells contrasts with the generally accepted idea that the “trisynaptic hippocampal circuit” is unidirectional, a pathway that relays information from EC to hippocampus (EC → DG → CA3 → CA1) and then to cortex (Amaral and Witter, 1989). Although the concept of a “back-projection” from area CA3 to the DG is not entirely new, it is generally considered to be indirect. Indeed, previous anatomical and physiological studies of mature GCs have provided evidence for a di-synaptic “back-projection” from CA3 to DG through hilar inhibitory interneurons and/or excitatory mossy cells (Scharfman, 2007). Our recent finding of a direct “back projection” is consistent with anatomical studies that showed that CA3 pyramidal cells axons can be found in the I-ML of the ventral DG (Li et al., 1994; Wittner et al., 2007). Indeed, the specificity of retroviral labeling for newborn neurons, combined with the selective TVA-EnvA retrograde tracing method, supports the notion of a direct “back-projection” from area CA3. However, it remains to be determined whether this connectivity is unique to newborn GCs and if so, what the potential functional consequences are. One could imagine a faster processing of information through a direct “back-projection,” which may support a specific role for new neurons in physiological processing of sequential memories (Lisman, 1999) and in pattern separation (Lisman, 2011).

Substantial input from septal cells was also observed at this time. Using immunohistochemistry for GABA and ChAT, we identified inputs to new GCs as cholinergic. Our observations indicate that newborn neurons receive direct robust input from septal cholinergic cells at 21 dpi (the starting point of our study, when new GCs consistently show spontaneous postsynaptic activity), suggesting that this innervation may be important during the maturation of new neurons. Indeed, it is quite possible that new GCs receive septal cholinergic innervation at earlier developmental stages (Ide et al., 2008). Previous research has shown that neurotoxic cholinergic forebrain lesions decrease cell proliferation and neurogenesis in the DG (Cooper-Kuhn et al., 2004; Mohapel et al., 2005), whereas activation of the cholinergic system with donezepil increases new cell survival (Kaneko et al., 2006). Both nicotinic (beta2, alpha7) and muscarinic (m1, m2, m4) acetylcholine receptors are present on the somata of immature GCs (Mohapel et al., 2005; Kaneko et al., 2006). Nicotine receptors have been implicated in cell proliferation and survival. Chronic nicotine administration (Abrous et al., 2002) and knockout of nicotinic beta2 receptors (Harrist et al., 2004) reduce hippocampal cell genesis while selective knockdown of alpha7 receptors in DG progenitors affects dendritic complexity and branching (Campbell et al., 2010). It has also been suggested that acetylcholine may have a modulating effect, by regulating excitability and network integration of newborn (olfactory bulb) neurons (Lin et al., 2010).

While rabies virus infection is almost exclusively restricted to neurons, there are electron microscopic reports of rare infection of glia cells (Matsumoto, 1963; Gosztonyi, 1994). It has been suggested that this may be indicative of inflammation associated with rabies virus administration (Marshel et al., 2010). However, rabies virus positive glial cells were associated with new neurons in the molecular layer of the DG (Vivar et al., 2012), as well as the olfactory bulb (Arenkiel et al., 2011). Immunocytochemical analysis showed that these cells express GFAP and are astrocytes. One possible explanation for an association between new GCs and astrocytes is the formation of a stem cell niche that influences the maturation of neuroblasts, similar to that observed in the subventricular zone (Lim and Alvarez-Buylla, 1999; Platel et al., 2010). Indeed, *in vitro* and *in vivo* experiments have shown that DG astrocytes support progenitor cell differentiation (Song et al., 2002; Barkho et al., 2006; Ashton et al., 2012). Interestingly, very few astrocytes were observed in the molecular layer at 3 weeks, however, an increase over time was observed, potentially associated with incremental spine density of the newborn neurons. Recent research has shown that astrocytes may express specific proteins important for the formation of excitatory synapses (Huang and Bergles, 2004; Allen et al., 2012). As the rabies virus appeared to “trace” these cells, this suggests that there may be synapse-like connections between new GCs and astrocytes. Indeed, astrocytes are considered to form synapse-like connections with the dendrites/spines of new GCs that may enhance synapse maturation and integration of new neurons (Toni and Sultan, 2011).

### Fourth week

At this time-point new GC processes continue to grow. Dendritic branching and protrusions, including mushroom spines, increase. The cells now have axosomatic, axodendritic and axospinous synapses (Zhao et al., 2006; Toni et al., 2007, 2008). Concurrently, mossy fibers have formed extensive contacts with area CA3 (Zhao et al., 2006; Faulkner et al., 2008; Ide et al., 2008; Toni et al., 2008). Glutamatergic and GABAergic (slow and fast) synaptic responses are detected, and enhanced intrinsic excitability is exhibited (Esposito et al., 2005). However, the new GCs still display immature characteristics compared to mature GCs, such as higher input resistance (R_in_) and smaller membrane capacitance (C_m_) (van Praag et al., 2002; Ambrogini et al., 2004; Esposito et al., 2005; Couillard-Despres et al., 2006; Mongiat et al., 2009). At this time-point, we observed that the new GCs continue receiving synaptic input from intra-hippocampal areas, including mature GCs, CA3 pyramidal cells and hilar cells (mossy cells and interneurons). Interestingly, intra-granular connectivity was substantially reduced as compared to 21 dpi. The reduction of this connectivity may be compensated for by gradual strengthening of distal cortical input (Vivar et al., 2012). Indeed, temporary overlap of intra-granular and cortical inputs may provide a mechanistic explanation for the transiently enhanced excitability and lower threshold for induction of long-term potentiation (LTP) in new GCs (Wang et al., 2000; Schmidt-Hieber et al., 2004; Ge et al., 2007) (Figure 4).

The EC is the major excitatory glutamatergic input to the DG. Projections arise from the LEC, considered to integrate novel environmental information, as well as from the MEC which contains grid cells with spatial specificity (Fyhn et al., 2004). LEC and MEC projections course through the lateral (LPP) and medial (MPP) perforant pathways, toward the outer (O-ML) and M-ML of the DG, respectively (Witter, 2007). Our trans-synaptic tracing approach revealed predominant LEC and PRH rather than MEC input to new GCs, as well as some input from dorsal caudo-medial entorhinal cortex (CEnt). Stimulation of the LPP and MPP evokes synaptic responses of larger amplitude in 1 month old GCs as compared to 3-week-old GCs, consistent with the strengthening of cortical input (Mongiat et al., 2009). Interestingly, stimulation of the MPP evokes synaptic responses in the new GCs even without substantial input from the MEC. There are several further questions that arise from this observation. First, is it possible that rabies virus may not trace well to the MEC and the observed response may be due to other glutamatergic inputs? This is unlikely, as upon labeling of both immature and mature GCs with lentivirus expressing TVA, GP, and GFP, trans-synaptic tracing is observed in the MEC, indicating that the rabies virus can reach this brain area (Vivar et al., 2012). Second, is it possible that axons from the LEC, usually confined to the O-ML (Witter, 2007), cross into the M-ML and mediate the synaptic response evoked by the stimulation of the M-ML in the new GCs? This remains to be determined. Third, recent research showed that selective optogenetic activation of the MEC evokes synaptic responses in 1 month old GCs (Kumamoto et al., 2012). However, could the elicited response be indirectly mediated via a polysynaptic pathway and/or by glutamatergic spillover (volume transmission) (Kullmann and Asztely, 1998)? Indeed, rabies virus propagation occurs at chemical synapses but not via cell-to-cell spread (volume transmission) (Ugolini, 1995; Tang et al., 1999). Consistent with this assumption, we were not able to see trans-synaptic tracing from ventral tegmental area or locus coereleus, two monoaminergic (dopaminergic and noradrenergic) areas, whose signaling is mainly mediated by volume transmission (Fuxe et al., 2007; Rice and Cragg, 2008), even though it has been described that dopamine modulates the activity of new GCs in the DG (Mu et al., 2011). Therefore, synaptic input from the MEC may be mediated by glutamate spillover in the M-ML, which may also play a role in the observed unique short-term plasticity of new GCs.

Evaluation of short-term plasticity in newborn and mature GCs revealed differences in integration of LEC and MEC inputs. It is well established that LEC and MEC projections to the DG exhibit distinctive physiological properties (McNaughton, 1980; Abraham and McNaughton, 1984). Stimulation of O-ML evokes paired-pulse facilitation (PPF), characteristic of LEC input, while stimulation of the M-ML evokes pair-pulse depression (PPD), characteristic of MEC input (McNaughton, 1980). Interestingly, at 1 month, new GCs exhibit PPF following M-ML stimulation instead of PPD (Vivar et al., 2012). Our findings are consistent with studies showing that stimulation of M-ML produced PPF in putative young GCs recorded from the inner GC layer (Wang et al., 2000) and in embryonic stem cell derived neurons transplanted into DG of hippocampal slice cultures (Benninger et al., 2003). However, the mechanisms underlying this differential response are as of yet unknown and may be associated with selective (segmental) dendritic spine maturation, whereby the medial portion of the cell could be preferentially activated by glutamate spillover and the outer portion by direct synaptic contacts.

Spine density in new GCs (6 weeks after retroviral injection) shows a progressive increment by branch order (Stone et al., 2011). Full maturation of the spines in the O-ML may take 6 months (Zhao et al., 2006; Toni et al., 2007). The medial portion may develop even more slowly and therefore contain a larger proportion immature dendritic spines or filopodia. At 30 dpi new GC filopodia are preferentially associated with boutons already synapsing on a dendritic spine (Toni et al., 2007). Development of these filopodia into spines may be regulated by neurotransmission (Harris, 1999), including glutamate spillover from active synapses (Kullmann and Asztely, 1998; Richards et al., 2005), which may in turn affect short-term plasticity. In addition, differential expression of glutamate receptors in young and mature neurons may play a role. AMPA receptor density is correlated with spine (Hall and Ghosh, 2008) and GC maturation (Hagihara et al., 2011). Furthermore, NR2B-containing NMDA receptors associated with enhanced synaptic plasticity in the new GCs (Ge et al., 2007) may be differentially expressed along the medial and outer portions of the dendritic tree. Indeed, structural and biophysical properties of the dendritic tree regulate synaptic input integration and neuronal function (Cline, 2001), and these appear to differ between newborn and mature GCs. Overall, the mechanisms underlying this differential short-term plasticity are as of yet unknown and future studies will be necessary to elucidate it.

The LEC appears to play an important role in the integration and function of the newborn GCs in the mature hippocampal network, similar to observations made during early postnatal development of new GCs, when EC axons preferentially make synapses onto distal dendritic GC segments (O-ML) (Frotscher et al., 2000; Förster et al., 2006). Indeed, LEC has been previously suggested to be important for adult neurogenesis (Froc et al., 2003; Shimazu et al., 2006). Shimazu et al. (2006) showed that deletion of the *NT-3* (Neurotrophin-3) gene results reduced adult neurogenesis and spatial memory function, and is associated with impaired DG LTP induced by stimulation of the LPP but not the MPP. Similarly, LPP stimulation failed to induce LTP in aged rats (Froc et al., 2003), which correlates with the observed decline in adult neurogenesis with aging (Seki and Arai, 1995). Moreover, LEC and MEC excitotoxic lesion experiments in young adult mice showed that the synaptic responses of retrovirally labeled newborn neurons to stimulation of either the LPP or MPP after LEC or MEC lesions were differentially affected. Both mature and new GCs exhibited impaired synaptic responses evoked by LPP stimulation after LEC lesion. However, only synaptic responses evoked in mature GCs by MPP stimulation were affected by MEC lesion (Vivar et al., 2012). Altogether these observations show that newborn GCs respond preferentially to LEC input, and suggest that synaptic connectivity/integration differs between mature and newborn GCs.

### Two and three months

At these time-points, new GCs continue receiving synaptic input from area CA3 pyramidal cells, hilar cells (mossy cells and interneurons) and distal cortex (PRH, LEC and CEnt) but not from mature GCs. Interestingly, hilar cell innervation increased from one to 3 months (Vivar et al., 2012). The majority of the hilar cells appear to be calretinin positive mossy cells (Blasco-Ibañez and Freund, 1997). Interestingly, recent research using a transgenic mouse model to selectively ablate these cells indicates that the net effect of mossy cells on GCs may be inhibitory rather than excitatory (Jinde et al., 2012). A smaller portion of the hilar cells were GABAergic interneurons, which expressed characteristic markers such as parvalbumin (PV), neuropeptide Y (NPY) and somatostatin (STT). In particular, NPY has been implicated in cell proliferation in the adult DG (Howell et al., 2005). In addition, recent research has shown that PV cells express TrkB receptors and may regulate newborn GC differentiation via a brain-derived neurotrophic factor (BDNF) dependent mechanism (Waterhouse et al., 2012). While interneurons are relatively few in number, these cells have an elaborate axonal arborization that forms synaptic contacts with multiple GCs (Freund and Buzsáki, 1996). The increased hilar input together with the reduction of intra-granular connectivity may lead to diminished excitability of new neurons observed during this time window (Ge et al., 2006). Even so, morphological plasticity of adult newborn neurons, for parameters such as dendritic branching and soma size, is reportedly greater than that of those born during development, at 2 and 4 months after retroviral labeling (Lemaire et al., 2012).

The distal cortical input from LEC/PRH became more robust over time. Analysis of the ratio of LEC/PRH cells to new GCs showed a strengthening of this synaptic input over 3 months after retroviral labeling. Trans-synaptic tracing from MEC was not obvious though cells were detected in the CEnt, which has both spatial and non-spatial memory processing functions (Sauvage et al., 2010). Electrophysiological recordings showed that at 60 dpi, newborn GCs still exhibit PPF. At 90 dpi, new neurons appear to begin to transition toward PPD (Vivar et al., 2012). Possibly, substantial input from MEC will develop upon complete maturation of the newborn GCs which has been suggested to take about 6 months (Toni et al., 2007). Overall, further research will be needed to elucidate how exactly the shift toward a more mature phenotype in new GCs occurs.

## Functional significance of adult neurogenesis

**Figure 5:**
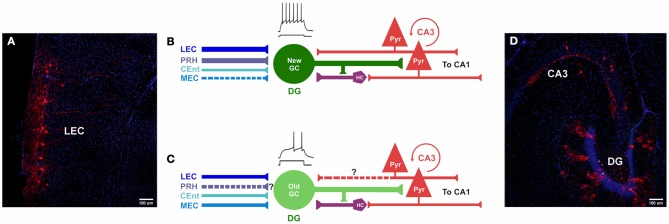
**Comparison of inputs to newborn and mature GCs. (A–D)** Photomicrographs and models of inputs, and their hypothesized relative synaptic strengths, onto newborn and mature GCs. **(A,D)** Photomicrographs of horizontal sections (40 μm) derived from a mouse injected with retrovirus, followed by rabies virus at 30 dpi (30 dpi retrovirus + 7 dpi rabies virus). **(A)** Retrograde tracing is observed in cortical layers II/III of LEC, traced cells express MCherry (red) and nuclei are labeled with DAPI (blue). **(B)** Newborn GCs receive synaptic input from PRH, LEC and CEnt rather than from MEC. The new neurons are highly excitable, their mossy fiber outputs enable firing in area CA3 pyramidal cells. New GCs receive both direct and indirect “back-projections” from area CA3. **(C)** Inputs from LEC, CEnt and MEC converge onto mature GCs. It is unknown (?) if they receive first-order afferents from PRH. Mature GCs are less excitable and may have a weaker impact on CA3. It is unknown (?) if mature GCs receive a direct “back-projection” from CA3, but indirect connectivity via hilar cells has been reported. **(D)** Horizontal section through the hippocampus shows double-labeling of hGFP and MCherry (“starter” cells, yellow) and traced cells in the DG and area CA3 (red). Nuclei are labeled with DAPI (blue). DG, dentate gyrus; PRH, Perirhinal Cortex; PYR, pyramidal cells; HC, hilar cells; LEC, Lateral entorhinal cortex; MEC, medial entorhinal cortex; CEnt, Caudo-medial entorhinal cortex. ? Unclear whether this is a direct input.

Research into the functional significance of adult neurogenesis has hitherto mainly focused on the DG. However, it should be considered that newborn GCs do not operate in isolation but rather are part of an elaborate neural circuitry important for learning and memory that originates in the EC. The flow of information from EC is generally considered to be propagated serially by excitatory synaptic transmission to DG → CA3 → CA1 and back to EC (Amaral and Witter, 1989). While it has become increasingly clear that there are recurrent networks between these areas (Lisman, 1999; Scharfman, 2007), for new GC neurons, this wiring diagram has further selective characteristics. Our recent work shows that newly born neurons receive preferential input from the LEC, PRH, and some innervation from CEnt, as well as a direct “back-projection” from area CA3 (Figures 4 and 5). This unique connectivity may have important implications for understanding the role of new DG neurons in memory function.

Information from the EC relays from two major cortical inputs, the MEC and LEC (Witter, 2007; van Cauter et al., 2012). The MEC, a region that contains grid cells, conveys highly specific spatial information to the hippocampus (“where”) (Fyhn et al., 2004; Hafting et al., 2005). In contrast, LEC is considered important for integration of sensory information about the environment, as well as for processing of novel object recognition and familiarity (“what”) (Myhrer, 1988; Zhu et al., 1995a,b; Hargreaves et al., 2005; Lisman, 2007; Murray et al., 2007; Deshmukh and Knierim, 2011). LEC receives non-spatial information mainly from the PRH (Insausti et al., 1997; Burwell, 2000). Evidence of a direct input from PRH to DG has been controversial (Liu and Bilkey, 1997; Canning and Leung, 1999). However, our recent work shows that newborn GCs receive direct input from the PRH (Vivar et al., 2012). The PRH is important for visual discrimination and novel objection recognition (Mumby and Pinel, 1994; Bussey et al., 1999; McTighe et al., 2010; Winters et al., 2010). PRH lesions lead to impairments in visual discrimination between closely related complex stimuli (Baxter and Murray, 2001; Bussey et al., 2003; Buckley, 2005; Bartko et al., 2007). In addition, aging related changes in the PRH in rodents have been implicated in the reduced ability to distinguish between objects with overlapping features (Burke et al., 2010, 2011). Pattern separation of perceptual sensory information in cortex may facilitate similar downstream mnemonic encoding processes. Indeed, the excitatory input from PRH/LEC may play an important role in enabling the DG to further process closely related events and locations and store them in memory. Preferential input to new GCs from PRH/LEC as compared to mature GCs, which are innervated by both LEC and MEC, suggests that newborn GCs may be more “specialized” in the processing of incoming environmental information than mature GCs.

Distinct storage of information is critical for minimizing memory overlap between closely similar stimuli and events. DG neurons outnumber EC cells resulting in sparse encoding (Treves and Rolls, 1992, 1994) considered to underlie pattern separation (Gilbert et al., 2001; Leutgeb et al., 2007; McHugh et al., 2007; Bakker et al., 2008; Yassa and Stark, 2011; Yassa et al., 2011; Schmidt et al., 2012; Hunsaker and Kesner, 2013), and newborn GCs may play an important role therein. Disruption of new neuron circuitry by PRH/LEC lesions (Vivar et al., 2012) as well as knockdown of adult neurogenesis by focal x-irradiation in the DG (Clelland et al., 2009) led to deficits in fine discrimination in the touchscreen task. In addition, in experiments in which neurogenesis is reduced followed by testing in contextual fear conditioning paradigms or the radial arm maze provide support for a role of new neurons in fine contextual discrimination (Guo et al., 2011; Nakashiba et al., 2012; Tronel et al., 2012). Furthermore, enhancement of neurogenesis (Creer et al., 2010; Sahay et al., 2011) results in improved pattern separation. This raises the possibility that new DG neurons may process contextual rather than spatial path integration information. Consistently, research showed that reduction of neurogenesis by x-irradiation selectively affected contextual fear conditioning, but not water maze or Y-maze learning (Saxe et al., 2006). In other behavioral studies, spatial memory was found to be impaired following knockdown of adult neurogenesis (Snyder et al., 2005; Imayoshi et al., 2008; Deng et al., 2009; Jessberger et al., 2009). However, tasks evaluated may rely both on external/contextual information (such as cues on the walls of a water maze room) as well as internal navigation strategies (traversing a maze requiring location of “self”), (Lisman, 2007). As reduction or silencing of new neurons also affects area CA3 function (Niibori et al., 2012), a brain area that receives direct input from MEC grid cells and is important for spatial path integration processes (Leutgeb et al., 2007), it may be difficult to determine the precise role of new neurons based on ablation studies.

Physiological research shows that DG cells have multiple place fields throughout an environment (Leutgeb et al., 2007). Interestingly, recent research suggests these could reflect the subpopulation of newly born GCs (Neunuebel and Knierim, 2012). Furthermore, computational modeling studies (de Almeida et al., 2009) indicate that the majority of DG cells are non-functional (Lisman, 2011), as well as that adult-born neurons may become redundant after 1 month of age and “retire” early (Alme et al., 2010), suggesting that young GCs may be the DG subpopulation of cells preferentially active in encoding incoming information. Indeed, transgenic mice in which output of old GCs (mossy fibers) to area CA3 was silenced had improved or normal pattern separation between similar contexts in fear conditioning paradigms. However, when young GCs were ablated, deficits in pattern separation were observed (Nakashiba et al., 2012), suggesting that new neurons are the main functional component. Thus, mossy fiber output to area CA3 may convey contextual rather than spatial position information and may arise from new rather than from mature, relatively silent, GCs. This output may be further modulated by the transiently enhanced plasticity of newborn as compared to mature GCs (Wang et al., 2000; Schmidt-Hieber et al., 2004; Ge et al., 2007).

Interestingly, newly born GCs receive direct innervation from area CA3 (Vivar et al., 2012). It is as of yet unknown whether mature GCs receive similar feedback or only indirect “back-projections” via mossy cells and inhibitory interneurons (Scharfman, 2007). While the functional significance of direct input from area CA3 to new GCs is unclear, this finding provides further support for the notion that recurrent circuits between the DG and area CA3 are important (Hunsaker et al., 2008; Myers and Scharfman, 2011; de Almeida et al., 2012). CA3 pyramidal cells have extensive collateral connections (Marr, 1971; Ishizuka et al., 1990; O'Reilly and McClelland, 1994; Rolls, 2010) which are considered to mediate pattern completion, a process where parts of objects or events are combined into a retrievable memory (Nakazawa et al., 2002; Hunsaker and Kesner, 2013). A back-projection can play a role in pattern separation (Myers and Scharfman, 2009, 2011), as there are fewer area CA3 than DG cells. Furthermore, the ability to make fine distinctions between closely related stimuli, such as the choice between adjacent arms in a radial arm maze (Clelland et al., 2009; Guo et al., 2011), may be supported by a component of spatial information from the MEC to area CA3, and then from area CA3 directly to newborn GCs. Behavioral studies where rats sustained area CA3 lesions suggest that the DG requires area CA3 for metric spatial detection (Hunsaker et al., 2008). In addition, monosynaptic area CA3 back-projections may be important for the correction of cumulative errors in episodic and spatial memory that could arise from serial uni-directional propagation of information in the network (Lisman, 1999; Lisman et al., 2005). If so, a feedback loop between the cells that may be most active in encoding new information (adult-generated GCs) and the CA3 auto-associative network may enhance efficiency and accuracy of memory storage.

Recent imaging studies in humans support the concept that pattern separation is mediated by circuitry consisting of EC, DG and CA3 (Yassa and Stark, 2011). It should be noted that this network is particularly susceptible to aging related changes (Bevilaqua et al., 2008). Especially the input from the EC via the perforant pathway is compromised with aging (Yassa et al., 2011). It is of interest that in Alzheimer's disease patients PRH and LEC are among the first cortical regions to be affected (Hyman et al., 1984; Braak and Braak, 1991). The close association between these brain regions and adult neurogenesis may open new windows for therapeutic interventions in humans. Further research, however, is needed to better understand the functional significance of adult neurogenesis within this memory circuit.

### Conflict of interest statement

The authors declare that the research was conducted in the absence of any commercial or financial relationships that could be construed as a potential conflict of interest.
